# ESEERP: Enhanced Smart Energy Efficient Routing Protocol for Internet of Things in Wireless Sensor Nodes

**DOI:** 10.3390/s22166109

**Published:** 2022-08-16

**Authors:** Roopali Dogra, Shalli Rani, Kavita, Jana Shafi, SeongKi Kim, Muhammad Fazal Ijaz

**Affiliations:** 1 Chitkara University Institute of Engineering and Technology, Chitkara University, Rajpura 140401, Punjab, India; 2 Department of Computer Science and Engineering, Chandigarh University, Mohali 140413, India; 3 Department of Computer Science, College of Arts and Science, Prince Sattam bin Abdul Aziz University, Wadi Ad Dawasir 11991, Saudi Arabia; 4 National Centre of Excellence in Software, Sangmyung University, Seoul 03016, Korea; 5 Department of Intelligent Mechatronics Engineering, Sejong University, Seoul 05006, Korea

**Keywords:** wireless sensor networks, Internet of Things, energy efficient, routing protocol, performance metrics

## Abstract

Wireless sensor networks (WSNs) have recently been viewed as the basic architecture that prepared the way for the Internet of Things (IoT) to arise. Nevertheless, when WSNs are linked with the IoT, a difficult issue arises due to excessive energy utilization in their nodes and short network longevity. As a result, energy constraints in sensor nodes, sensor data sharing and routing protocols are the fundamental topics in WSN. This research presents an enhanced smart-energy-efficient routing protocol (ESEERP) technique that extends the lifetime of the network and improves its connection to meet the aforementioned deficiencies. It selects the Cluster Head (CH) depending on an efficient optimization method derived from several purposes. It aids in the reduction of sleepy sensor nodes and decreases energy utilization. A Sail Fish Optimizer (SFO) is used to find an appropriate route to the sink node for data transfer following CH selection. Regarding energy utilization, bandwidth, packet delivery ratio and network longevity, the proposed methodology is mathematically studied, and the results have been compared to identical current approaches such as a Genetic algorithm (GA), Ant Lion optimization (ALO) and Particle Swarm Optimization (PSO). The simulation shows that in the proposed approach for the longevity of the network, there are 3500 rounds; energy utilization achieves a maximum of 0.5 Joules; bandwidth transmits the data at the rate of 0.52 MBPS; the packet delivery ratio (PDR) is at the rate of 96% for 500 nodes, respectively.

## 1. Introduction

A WSN is made up of several sensor nodes that are connected in an ad hoc and transitory fashion to detect data and transmit it to a centralized location known as a base station (BS) or sink node [1]. Sensor nodes face several limitations in terms of processing power, energy resources, memory, and storage, which are not present in standard wireless networks. The main purpose of WSNs is to arbitrarily disperse sensor nodes in unsupervised places and enable wireless communication. Various routing algorithms have been designed in conventional networks to improve network performance and growth in terms of delay and average throughput. Conventional algorithms are not ideal for IoT applications because the structure of the IoT is complicated and more dynamic in an unstable wireless environment. Numerous information and network vulnerabilities happen as a consequence of the rapid expansion of IoT systems, obstructing their growth. In general [2], data aggregation and forwarding techniques can be divided into two groups. First, there is a framework technique which gathers sensor information without the need for a fixed framework and conducts data aggregation utilizing incomplete data. Second, there is a framework technique that splits the network field into various clusters. A single local data-aggregator node, which gathers data from all its connected members and executes aggregation operations, exists within each region. It then sends the consolidated data to the sink node over a pre-established communications network. Sensor nodes operate independently in most WSN applications and are vulnerable to a variety of security risks.

**Figure 1:**
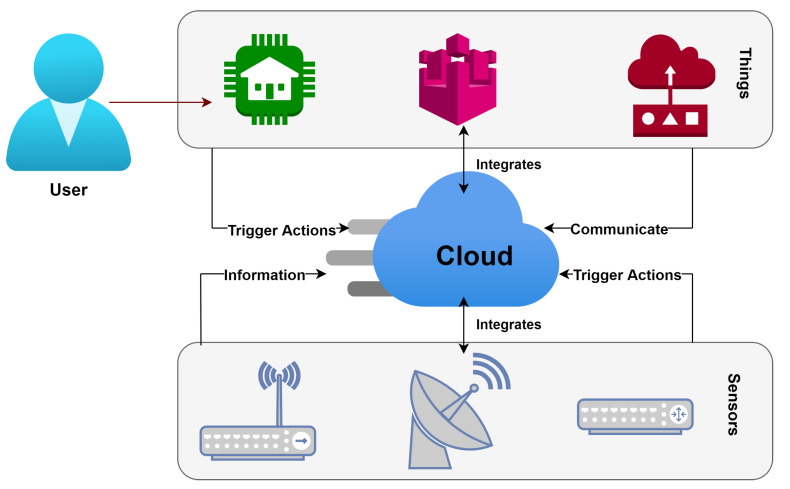
IoT based WSN architecture.

A WSN is a network of several nodes which senses the environment, manipulates the acquired data and thereafter transmits and receives the sensed information in a particular network [3]. The fact that these sensor nodes have low maintenance costs, are self-configurable and have an abundance of applications in which they play a significant role have made them a favorite for every sector of human life. If the above conditions are met, A WSN facilitates us in applications in different sectors such as agriculture, military operations, health monitoring and environmental sensing [4]. The basic architecture of IoT-WSN is demonstrated in Figure 1. The sensor nodes sense the data and transmit them to the base station or sink. From there, the data are forwarded to the user via the Internet to perform further necessary operations. Each of the sensor nodes used in a WSN is resource-constrained due to its small-sized configuration [5]. The main components of any sensor node include a processing unit for different computations, a transceiver for transmission and reception of data, a sensing unit comprising sensor and ADC and a power unit for providing a power source to the sensor node.

### 1.1. Significant Approaches towards Acquiring Energy Efficiency

The above discussion shows that it is not plausible to replenish the motes for some applications such as structural health monitoring, environment monitoring, transportation systems and precision agriculture [6] because of the number of motes, the maintenance cost involved or the remote areas where human intervention is not possible. In a nutshell, it is clear that it is the application of a WSN that decides the fate of a node in the context of its energy consumption. By bearing that in mind, the different approaches for achieving energy efficiency are listed and explained below [7].

**Wireless Communication Optimization:** Radio characteristics decide the energy consumption of nodes. If a node is communicating with the other node, then many factors such as its control on transmission power and different modulation schemes incorporated play a significant role. Cooperative communication and cognitive radio also balance the energy consumption by focusing on the quality of data transmission. These factors are explained below.

**Transmission Power Control:** Wireless communication optimization considers transmission power control (TPC). The maintenance of the level of transmission power of a node at a significant specific level with optimized energy consumption is TPC [8]. The energy efficiency of a WSN is directly dependent on the radio characteristics of the wireless sensor nodes [9]. When the data transmission is high in the nearest and known path due to the optimized cluster head, then it guarantees much better throughput. However, the selection of the optimized cluster head and searching for the best route lead to a rapid reduction in battery power level [10].

**Research Gap:** Due to the application-specific nature of a WSN, the development of TPC schemes becomes a daunting task. There are two prominent factors, distance and the quality of the link, that decide the required level of transmission power to reach the receiver. Link quality has some dependence on physical barriers such as environmental conditions and physical obstacles in the path of transmission among nodes. TPC concerning the link quality among nodes is an energy- efficient approach as it selects the minimum power needed to maintain a qualitative link; most TPC schemes are proposed for single-hop communication. TPC is handled with two processes, i.e., discovery of neighboring nodes and setting up a feedback or acknowledgment system for the nodes that have received messages. There are several thresholds defined for deciding the link quality and handling the neighboring tables that store the information about the neighboring nodes.

**Cooperative Communication:** In the recent years of technological advancements, MIMO (multiple input multiple outputs) antenna technologies have outperformed the traditional wireless technologies [11]. While it can be implemented in resource-constrained wireless sensor nodes, the installation of an additional antenna would lead to additional space requirements on the node platform. The circuit becomes complex, and the cost of sensor nodes is enhanced enormously [12]. Consequently, dual-antenna approach implementation becomes quixotic. To resolve this issue, individual single antenna nodes are made to cooperate with the other nodes; hence a cooperative MIMO scheme is implemented. When several such nodes are assembled to form such a structure, it is termed a virtual MIMO. Cooperative communication helps in acquiring energy efficiency in a way that ensures reliable communication among the nodes and the base station [9]. When multiple nodes send data by forming a MIMO structure, it not only speeds the data delivery but also helps in reducing the packet drop in the network.

**Research Gap:** The current research demonstrates the cooperative communication framework implemented in the network. It emphasizes the way the cluster head nodes build up communication among the cooperative nodes in the network to efficiently utilize energy resources in the network.

**Energy Efficient Cognitive Radio:** A cognitive radio (CR) is described as a radio that adapts its transmitter parameters to the environment in which it operates [13]. A CR is a smart radio that can dynamically select a wireless communication channel and adapt its parameters of transmission and reception accordingly [11]. The underlying software-defined radio (SDR) technology is anticipated to produce fully reconfigurable wireless transceivers that automatically adapt their communication parameters to network requirements, enhancing context awareness [14]. However, CR needs considerable energy consumption compared to standard appliances due to the enhanced complexity of fresh and complex features [15]. Constructing an energy-efficient cognitive radio WSN is the main challenge in the smart use of battery energy in this context. Recent cognitive radio studies focus on transmitter energy control, residual energy-based channel allocation and network coding and CR combination. Open research problems include the creation of MAC, routing or clustering protocols that take advantage of cognitive radio possibilities.

### 1.2. Deployment Strategies

Sensors can be put either deterministically or randomly in a region of concern [16]. Deployment system selection is extremely dependent on the type of sensors, the implementation and the environment in which the sensors will function. Controlled node deployment is feasible and often necessary when sensors are costly or when their position affects their operation considerably. Such situations involve populating an area with extremely accurate seismic nodes, WSN apps underwater and video and image sensors. On the other hand, random node allocation is the only viable alternative in some apps.

- Controlled node deployment: Controlled deployment is usually pursued in indoor applications of WSNs [17].
- Random sensor deployment: Randomized positioning of sensors is often the only choice. For instance, in WSN apps in battle recognition tasks, disaster recovery and forest fire detection, sensor deterministic deployment is highly dangerous and/or unfeasible. It is commonly anticipated that helicopters, grenade launchers or clustered bombs will drop sensors. Such deployment implies random sensor spread although to some extent, node density can be monitored. While substantial progress has been made in investigating node positioning optimization in WSNs, many challenges remain [10].

**Sleep Scheduling Mechanism:** Sleep scheduling implies the proportion of the wake-up moment in a predefined period to the complete duration of the period [18]. For instance, if one node remains active for 0.1s and sleeps for 0.9s for a period of 1s, then the ratio is 0.1. Sleep planning processes for WSNs can usually be categorized into three classifications.

- Synchronous Schemes: In synchronous systems such as S-MAC and T-MAC, sleeping nodes wake up at the same moment to interact with each other regularly, which implies that the network must maintain global synchronization.
- Semi-Synchronous Schemes: Sensor nodes are usually grouped into clusters concerning semi-synchronous systems. Sensor nodes wake up or go to sleep simultaneously in the same cluster, but clusters behave asynchronously with others.
- Asynchronous Schemes: Each sensor node has its wake-up and sleep timetable in terms of asynchronous systems.

The purpose of a WSN sleep schedule is to save the WSN’s energy consumption. Here, we list some possible instructions [19].

- Mobility for relay and mobility WSNs: The nodes consume energy which further leads to an energy hole problem. The mitigation for this problem is possible with the help of a mobile relay and sink. Similar to a mobile robot, the mobile relay or mobile sink can move around to collect data, providing a nice trade-off between energy consumption, latency and delay in shipment.
- Clustering process for WSNs: For semi-synchronous sleep scheduling schemes, the network is generally divided into several clusters, and cluster heads are responsible for interacting with other clusters. The cluster heads may need to consume more energy compared to normal sensor nodes [20].

### 1.3. Clustering for Smart Energy Efficiency

**Figure 2:**
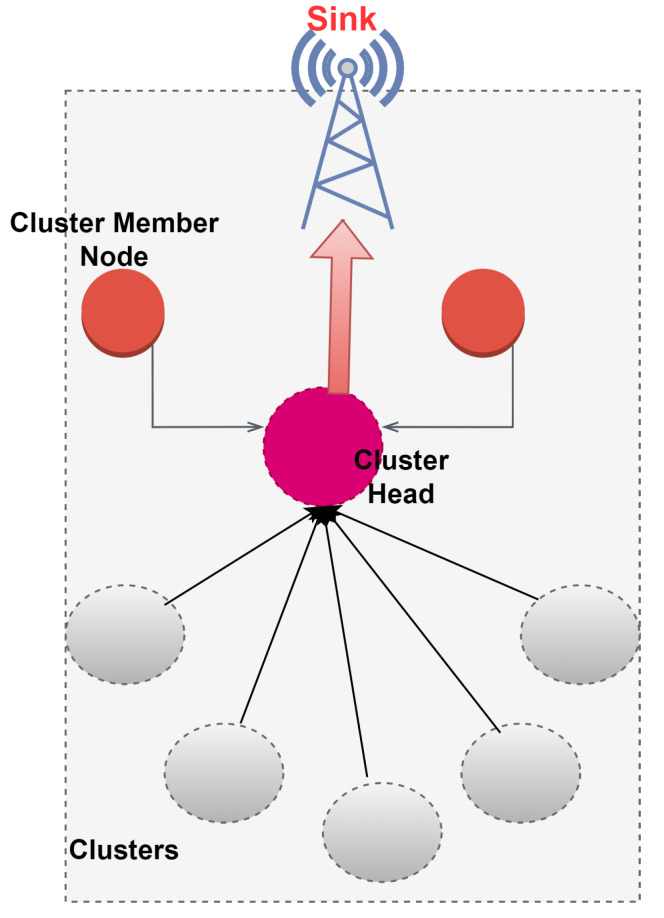
Clustering illustration.

Numerous routing techniques that tend to enhance the energy efficiency of the network have been reported. Clustering proves to be promising in preserving the energy of the nodes. It is defined as the grouping of sensor nodes to form a cluster as shown in Figure 2. Every cluster has one cluster head (CH) that assembles the data from the cluster, aggregates it and then forwards it to the next CH or the base station depending upon the routing topology employed. The main concern when adopting clustering topology is the selection of the CH. It could be based on residual energy and the distance of the node from the sink. However, the problem of selecting the most appropriate CH still exists. The important concern of energy efficiency is being addressed by the researchers in WSN [20]. Homogeneity does not exist practically, and it is observed that heterogeneity in sensor nodes brings more energy balance to the network. Therefore, this paper focuses on heterogeneous routing protocols. It is worth mentioning that LEACH (Low Energy Adaptive Clustering Hierarchy) was the first cluster-based routing protocol that inspired other protocols to incorporate heterogeneity in their work [21].

The first protocol to define heterogeneity in routing was the SEP (Stable Election Protocol) [19]. It used energy heterogeneity at two levels. The CH selection in the SEP was based on the weighted probability for selecting any node as CH. However, it failed for more than two levels of heterogeneity. Since the development of the SEP, various routing protocols have been presented that mostly focused on CH selection. DDEEC (Design of Distributed Energy Efficient Clustering) improved the CH selection method by avoiding the penalization of high energy nodes [20]. It used residual energy for CH selection. After the advancements in different routing strategies, energy heterogeneity at four levels was introduced by the BEENISH (Balanced Energy Efficient Network Integrated Super Heterogeneous) protocol for wireless sensor networks. However, the BEENISH suffered from the drawback of the penalization of higher energy nodes in the process of CH selection [22]. MRA (Multiple gateway-based Routing Architecture) introduced the novel method of data collection for harsh environment monitoring. In this, the authors utilized four gateways around each periphery of the network [23]. It aimed for the early detection in harsh environment monitoring. The EEZECR (Energy Efficient Zone-based Energy Clustering Routing) protocol utilized a zone divisional method for performing routing in a WSN. It proved to be energy efficient, but it suffered from the complexities in the network [24]. Several other attempts have been made to enhance the energy efficiency of routing protocols. One proposed a unique way of saving the network from heavily burdening the relay nodes which is also termed a hot-spot problem [25]. In this work, the aforementioned issues are addressed by selecting a CH based on the residual energy, distance factor and, most importantly, the network energy. The topology remains conventional where the routing scheme does not form any sector. Rather, unequal clustering that saves a huge number of overheads is followed.

### 1.4. Problem Definition

The energy consumption scenarios discussed in the previous section demonstrate that the communication of a node drains almost all of the energy. Unlike the sensing and computational task which consumes very little energy, the transmission and reception of data use tremendous amounts of energy. The motivation behind highlighting the energy efficiency of a WSN is the inevitable energy drainage of a resource-limited sensor node [26,27]. The WSN is entirely application-specific; therefore, its design take energy consumption into account. Moreover, any system that provides precision in detecting any surrounding attribute with the same amount of energy consumption is termed energy efficient. Consequently, energy efficiency proves to be a trade-off between various performance parameters such as bandwidth, PDR, network longevity and energy utilization where a designer has to decide the specific requirements. So, it all depends on the application where the sensor nodes are to serve, and accordingly, the network longevity is achieved by keeping the network energy efficient. The sole objective of network longevity does matter for those applications where a delay is unacceptable, such as forest fire detection and other catastrophic events. In those applications, energy efficiency must be obtained in the form of reducing the delay in data delivery for each unit of energy consumption. Similarly, for some applications, the QoS (Quality of Service) parameters are of paramount importance; their network longevity is a secondary concern. Therefore, in this paper, the primary emphasis is on highlighting the stumbling blocks in acquiring energy efficiency and providing some measures to curb energy consumption as much as possible. There have been various surveys [6] criticizing the energy efficiency WSNs; however, the simulation analysis along with an optimum solution to attain smart energy efficiency is still unexamined. This motivates us to spotlight the energy efficiency aspects along with simulation analysis of the proposed strategies to achieve a smart energy-efficient WSN.

The main gap in the existing literature is that the number of nodes are static for every round that does not ensure the number of acquired cluster heads will maximize the lifetime of network. In our proposed protocol, the number of nodes varies for every round which increases the efficiency and lifetime of the network.

### 1.5. Major Contributions

The key contributions of this paper are summarized as:

1. Data aggregation, multi-objective-based cluster head selection and optimization-based path selection for transmitting data are the three primary processes in the framework network for smart-energy-routing algorithms that are developed.
2. The model for a multi-path network is deployed where the distance D between the transmitter and receiver is more than the threshold value, which is the distance square for the dissipation of radio energy for the multipath model.
3. The cluster head selection method is chosen for every round to achieve network longetivity and energy efficiency.
4. The performance is evaluated based on the metrics and compared with baseline algorithms (PSO, GA, ALO) with the proposed methodology.

## 2. Methodology

**Figure 3:**
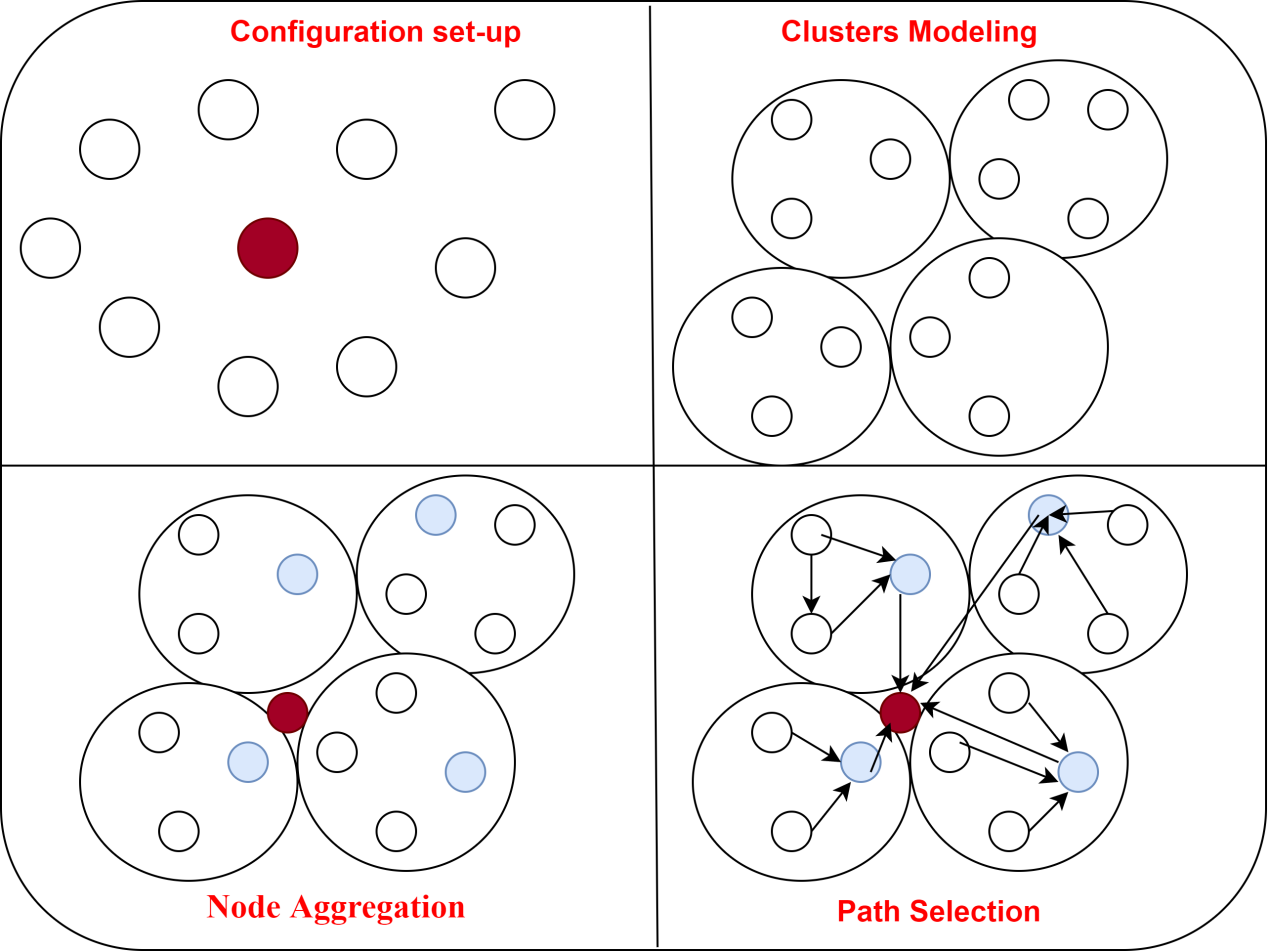
Proposed phases for energy routing protocol.

Data aggregation, multi-objective-based cluster-head selection and optimization-based path selection for transmitting data are the three primary processes in the framework network for smart energy routing algorithms. It concentrates on the issue of traffic congestion around the sink. Configuration, energy modeling, node aggregation and path selection are the four stages of the process. The sensor network development and installation are explained in the configuration step. Transferring data via the nodes and an analysis of energy use are part of the energy modeling. The node aggregation and cluster-head selection describe how to classify the nodes in the network and create clusters. The SFO method will be used to determine the best path for transferring data during the path selection stage. The proposed flowchart is shown in Figure 3:

Assume a network as a graph G = V, E, where V denotes the sink node or base station, and E denotes the link required for transmission among sensor nodes. We make several assumptions for the network. For randomized sensor placement, a two-dimensional Euclidean approach is used. A non-rechargeable power supply is included with each sensor node; being deployed, sensor nodes are unable to change their placement. The dissipation of energy for radio is utilized. When the recipient of radio electronics uses energy, simultaneously, the transmitter amplifies and transmits the energy. The model for the multi-path is deployed where the distance D between the transmitter and the receiver is more than the threshold value, which is the distance square for the dissipation of radio energy for the multipath model. However, the m-bit transmission of packets for energy utilization $W_{x}$ is shown by Equation (1),

When

$$distance<g_{0},$$

$$W_{x}=m\left(W_{ec}+W_{frs}*distance^{2}\right)$$

and otherwise,

(1) $$W_{x}=m\left(W_{ec}+W_{mpf}*distance^{4}\right)$$

Algorithm 1 of the routing protocol is explained to show the mechanism of how the cluster head is transmitted to the base station.
**Algorithm 1:** Algorithm for proposed route1:**procedure**Begin:2:   **For each and every round of CH**3:   Choose the optimal number of CH’s4:   Organize the clusters5:   **For each and every CH**6:   Choose the cluster coaggregation based on the remaining energy7:   **For each and every coaggregation**8:   Merge and forward the data to cluster head9:   **endfor**10:    CH forwards to the base station11:    **endfor**12:    **endfor**

**Figure 4:**
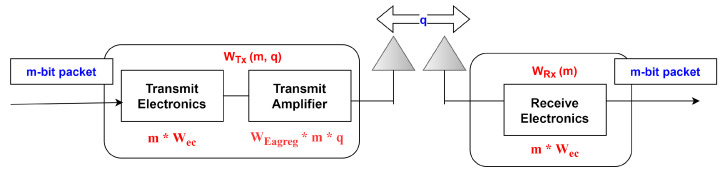
Model for dissipation of energy.

As shown in Figure 4, the electronic circuit of the sensor nodes requires energy $W_{ec}$ to recognize the energy amplified in either free space or the multipath model between the transmitter and receiver. It is bit-rate tolerant, and the model is shown as $W_{frs}$ ∗ $distance^{2}$ and $W_{mpf}$ ∗ $distance^{4}$. $W_{frs}$ and $W_{mpf}$ are the energy requisites for transmitting a bit into the free space employed through the multipath channel with g distance between the transmitter and receiver, whereas distance 0 is the distance calculated by the use of the threshold in Equation (2).

(2) $$distance_{0}=\frac{\sqrt{W_{frs}}}{\sqrt{W_{mpf}}}$$

The utilization of energy for packets received of m bits is represented by Equation (3):

(3) $$W_{rec}=m*W_{ec}$$

For the data aggregation, the energy used is represented by Equation (4):

(4) $$W_{agreg}=W_{Eagreg}*m*q$$

where the bits acquired in a packet are shown as “*m*”, the number of messages is shown as “*q*”, and the energy utilized for one-bit aggregation is shown as $W_{agreg}$.

### Cluster Head Selection

A cluster head with more than one node eligible to act as nodes are named “cluster congregation (CGA)”. The CGAs are in charge of acquiring and consolidating data from the members of the clusters before sending them on to the cluster heads using a hybrid MAC protocol. To transfer sensed data to its CGA (low-level MAC), the members of cluster nodes use a CSMA/CA protocol to acquire the channel. Every CGA, on the other hand, sends the gathered information to its CH during its given TDMA session (high-level MAC).

Each member of the cluster turns on its radio communication modules in the steady-state period to transmit the perceived data to the appropriate CGA. As a result, the nodes would only be activated throughout their operating hours, and they will otherwise hibernate. The data are aggregated, compressed and forwarded to the cluster heads by the CGAs. As a result, the intra-cluster transmission mechanism is multi-hopping [28]. Moreover, every CH sends the data it receives to the base stations. The cluster heads transmit END round notifications to the participants after the base station receives all the data. For each round, the total energy used by the cluster head is illustrated in Equation (5). The $W_{R}$ denotes the total energy drawn by a CH in each round; $W_{SCH}$ shows the energy utilized at the CH selection step; $W_{ACH}$ represents the energy utilized to broadcast the CH advertisement; $W_{RCJ}$ shows the energy utilized to acquire the joint request from non-CH nodes; $W_{RC}$ represents the energy utilized to acquire the sensed data from member of the cluster; $W_{SC}$ is energy utilized to send the aggregated data from the CH to the base station.

(5) $$W_{R}=W_{SCH}+W_{ACH}+W_{RCJ}+W_{RC}+W_{SC}$$

I $W_{R}$ depicts the $W_{SCH}\left(o,t\right)=\left(\left(o_{c}/\alpha \right)*\left(W_{EC}+\epsilon_{frs}distance^{2}tobasestation\right)\right)$

where $o_{c}$ represents the size of the control packet

$W_{ACH}\left(o,t\right)=o_{c}*W_{elec}$;
$W_{RCJ}\left(o\right)=N_{cm}*o_{c}*W_{ele}$;
$W_{RC}\left(o\right)=g*\left(N_{crao}*o_{distance}*W_{elec}\right)$;
$W_{SC}\left(o,t\right)=\left(o_{distance}/\alpha \right)*\left(\epsilon_{frs}distance^{2}tobasestation\right)$.

On either side, the overall energy utilized by the members of the clusters and congregations of each cluster node in every round $\left(W_{MR}\right)$$\left(W_{GRA}\right)$ can be computed as described in Equation (6). $W_{MR}$ denotes the total energy utilized by a cluster member in each round; $W_{MoJ}$ shows the energy utilized to transmit a joint message to the CH; $W_{MO}$ denotes the energy utilized to send the sensed data from a member of the cluster to the cluster head.

(6) $$W_{MR}=W_{MtA}+W_{MoJ}+W_{MO}$$

and

(7) $$W_{GT}=W_{GsP}+W_{GR}+W_{GaP}+W_{GS}$$

$W_{GT}$ signifies the energy the cluster congregation uses; $W_{GsP}$ shows the energy utilized to broadcast the cluster congregation advertisement; $W_{GaP}$ denotes the energy utilized by the process of data aggregation. Based on the utilization of energy, the behavior of the node can be depicted in the proposed protocol which assumes that for every round the node $n_{a}$ should take one of the three roles: cluster head, member of the cluster or congregation of clusters. Therefore, the evaluation of the utilization of energy W for node $n_{a}$ is based on the role over every round r1.

The fitness function for all the clusters for every round in the SFO is described with the objective function: CH fitness value = F1(CH). There are different objectives in preventing failure of the sink node among which is evaluating the bandwidth of the CH which includes the maximal number of data sent to the base station from CH.

## 3. Enhanced Cluster-Head Selection in a WSN for IoT

The integrity of the routing protocol has a direct impact on network longevity and efficiency, whereas a robust topology strategy depends on a comprehensive evaluation methodology [28,29]. Depending upon the various characteristics, various indicators are required to compute the network lifetime and smart energy efficiency:

- **Network Coverage:** Coverage is a metric for WSN service quality that focuses on the preliminary node deployments coverage rate and if these nodes can obtain signals from the ROI entirely and properly.
- **Network Connectivity:** Because sensor networks are typically big in scale, connection ensures that data collected by sensors can be transferred to sink nodes [24].
- **Network Longevity:** The timeframe from the inception of the network to when the percentage of dead nodes reaches a certain threshold is referred to as the lifetime of the network.

The Algorithm: 2 for the selection of cluster heads is:
**Algorithm 2:** Algorithm for selection of cluster head     **Input:** Collection of sensor nodes C = $c_{1},c_{2},c_{3}$     **Output:** Choosing the best sensor node as CH1:**procedure**Begin:2:    **for** i=1;i<=n;i++3:    **while** choosing the CH4:    **For** every sensor node c_i5:    Compute proximity $N_{prox}$ = $\frac{1}{N}\sum_{i=1}^{1-N_{T}}d\left(n,i\right)$6:    **if** ($N_{prox}$ is maximized)7:    choose the sensor node c_i8:    **else**9:    Reject the sensor node c_i

The enhanced cluster-head selection procedure consists of the following specific steps:

1. The network is being configured with a base station that can obtain the coordinates of all sensor nodes in the sensing area as well as their remaining energy.
2. The monitoring region is separated into several clusters using a Voronoi diagram, and a probabilistic model for perception is developed. The attenuation probabilistic algorithm is used to choose duplicate network nodes, and these nodes are considered the first type of hibernating cluster-head node [16].
3. When one of the cluster-head nodes dies, another duplicate node takes over as cluster head. If the death node is a current common node, another duplicate node awakens from sleep to become a normal node.
4. If the first type of cluster head nodes expired, the survival time evaluation method could be used to determine the remaining network aggregate energy [30]. Calculated by the ratio of the remaining energy to available energy of nodes in the network, we choose the next class of cluster-head nodes.

### Transmitting Data to the Base Station

The process of sending data from each cluster head to the base station has been consolidated [31]. The SFO is used to locate the best possible path for transferring data over the network. Initializing the route choosing method, transmitting data and modifying the route are the three main steps of the SFO-based routing procedure [32]. SFO is regarded as a population-based metaheuristic algorithm. The sailfish location is designated as a problem’s variable in the solution space, and a candidate’s solutions are regarded as sailfish. At the solution space, the population is created at random. Sailfish can explore in a hyper-three, two- or one-dimensional space depending on the location of the vectors.

In the initializing phase, based on the coordinates of vectors, the SFO finds the solution in a hyper-dimension. By recognizing the best adjacent cluster head, the route is chosen based on its fitness value. Later, the recognized best adjacent cluster head is replaced with another cluster head and recognizes the best route [33]. The procedure for transmitting the data will be started once the best route is recognized amongst the numerous routes. The utilization of energy for data transmission of the packets is sent to the base station which may cause the death of the cluster head or minimization in its energy [34].

The main advantage of the SFO algorithm is that it provides the customization of parameter configurations which enhances the performance compared to the default setting of the parameters.

## 4. Performance Analysis and Evaluation

**Figure 5:**
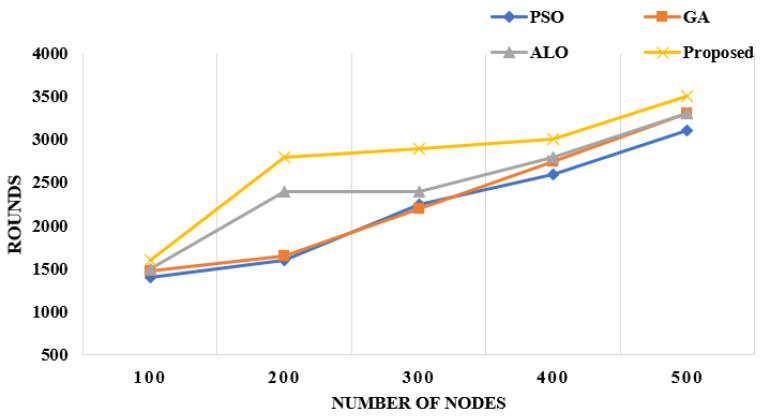
Network lifetime with the number of nodes [10,11,15].

The evaluation of performance is implemented in MATLAB, and the comparison is made with existing algorithms such as GA [9], ALO [11] and PSO [8] in terms of energy efficiency, bandwidth, packet delivery ratio and network longevity [35,36,37]. With GA, the structure of the network differs with every round based on the features of the nodes; ALO maximizes the energy utilization and distance, having a minimized response time; PSO calculates the optimal set of cluster heads which maximizes the efficiency of energy and coverage of the network. The simulations carried out during the implementation are shown in Table 1:

**Network Longevity:** It indicates how long or for how many rounds the network can operate. It refers to the number of rounds after which the nodes in the field will expire whilst completing their jobs. In Figure 5, the number of nodes is 100 to 500. It shows the proposed framework achieves more longevity compared to the other existing approaches. The proposed framework delivered the packets successfully with the 3500 rounds for 500 nodes based on the number of rounds; for every round, longevity of the network increases.

**Figure 6:**
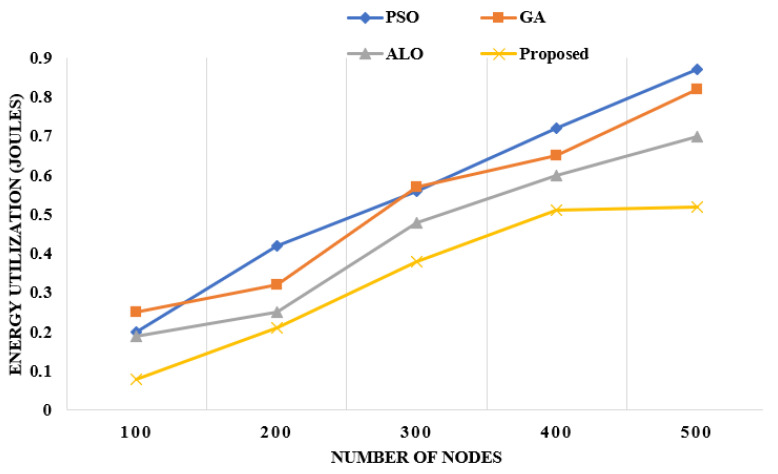
Energy utilization with regard to number of nodes [10,11,15].

**Energy Efficiency:** This is the amount of energy utilized by the sensor nodes in the network. As shown in Figure 6, the proposed framework has utilized the energy with a maximum of 0.5 Joules with 500 nodes, whereas the other existing approaches utilize more energy than the proposed approach.

**Figure 7:**
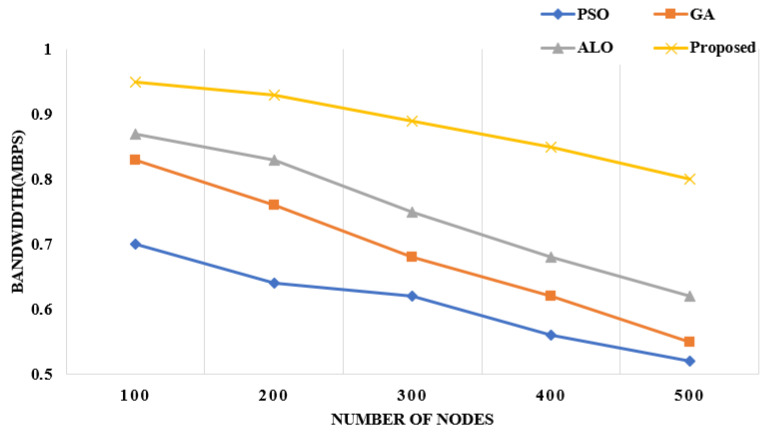
Bandwidth with regard to number of nodes [10,11,15].

**Bandwidth:** It represents the amount of data transmitted to the base station with several nodes. It is denoted as the number of packets transmitted *size of the packet which is divided by the time taken by the number of nodes. As shown in Figure 7, the comparison amongst the existing approaches is depicted in the figure bandwidth w.r.t number of nodes [31]. It shows that the proposed framework with 500 nodes transmits data at the rate of 0.52 MBPS compared to the other approaches.

**Figure 8:**
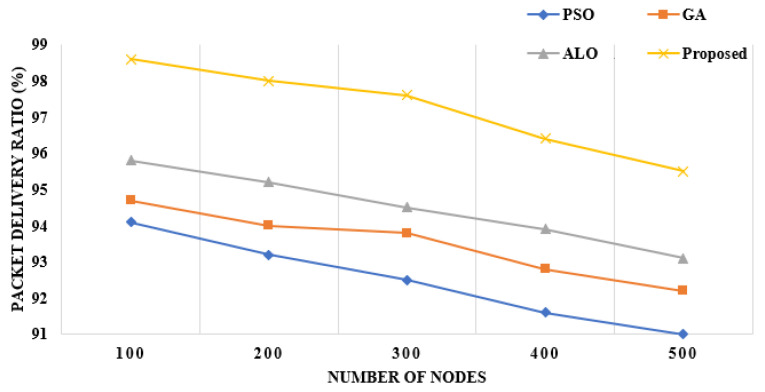
Packet delivery ratio with regard to number of nodes [10,11,15].

**Packet Delivery Ratio:** It expresses the ratio of data packets effectively transmitted to the base station with various numbers of nodes. In Figure 8, the proposed framework shows that it has successfully transmitted the packets with 500 nodes at the rate of 96% compared to the other approaches. The figure shows that as the number of nodes are increasing, the ratio of PDR decreases.

## 5. Conclusions

In this study, a novel solution for IoTs in WSNs, an ESEERP protocol, is designed and developed. It solves the issue of sensor nodes near the base station having a short lifespan. The reduced life issue comes as a result of heavy traffic from various cluster heads to the sink. The ESEERP employs an energy-efficient cluster head selection algorithm that considers a variety of factors such as distance, cost, remaining energy and scope. The SFO is used to choose the best route from the cluster head to the sink node. The ESEERP efficiency is conducted to evaluate the performance of existing optimization-based routing methods. The results have shown that with 500 nodes, the ESEERP endured for roughly 3500 rounds, which is better than the existing ones. The limitations of the proposed approach are that there is unequal consumption of nodes that hugely affect lifetime of the network, and overhead maximizes as the threshold-based functions are maximized. In future work, we will examine the cluster optimization is to be done to improve the efficiency and achieve balanced energy consumption.

## Figures and Tables

**Table 1:** Simulation environment.

QoS Metrics	Values Deployed
Area	100 m × 100 m
Number of nodes	100 to 500
Number of Clusters	Varies
Energy of nodes	1 Joule
Network energy	Based on the number of nodes
Size of packet	1250 bytes
Bandwidth	1 Mbps

## Data Availability

Not applicable.
